# Supplementary figures and images for: Schistosoma haematobium infection is associated with lower serum cholesterol levels and improved lipid profile in overweight/obese individuals
Source: PLoS Negl Trop Dis. 2020 Jul 2;14(7):e0008464. doi: 10.1371/journal.pntd.0008464 (PMC7363109; doi:10.1371/journal.pntd.0008464)

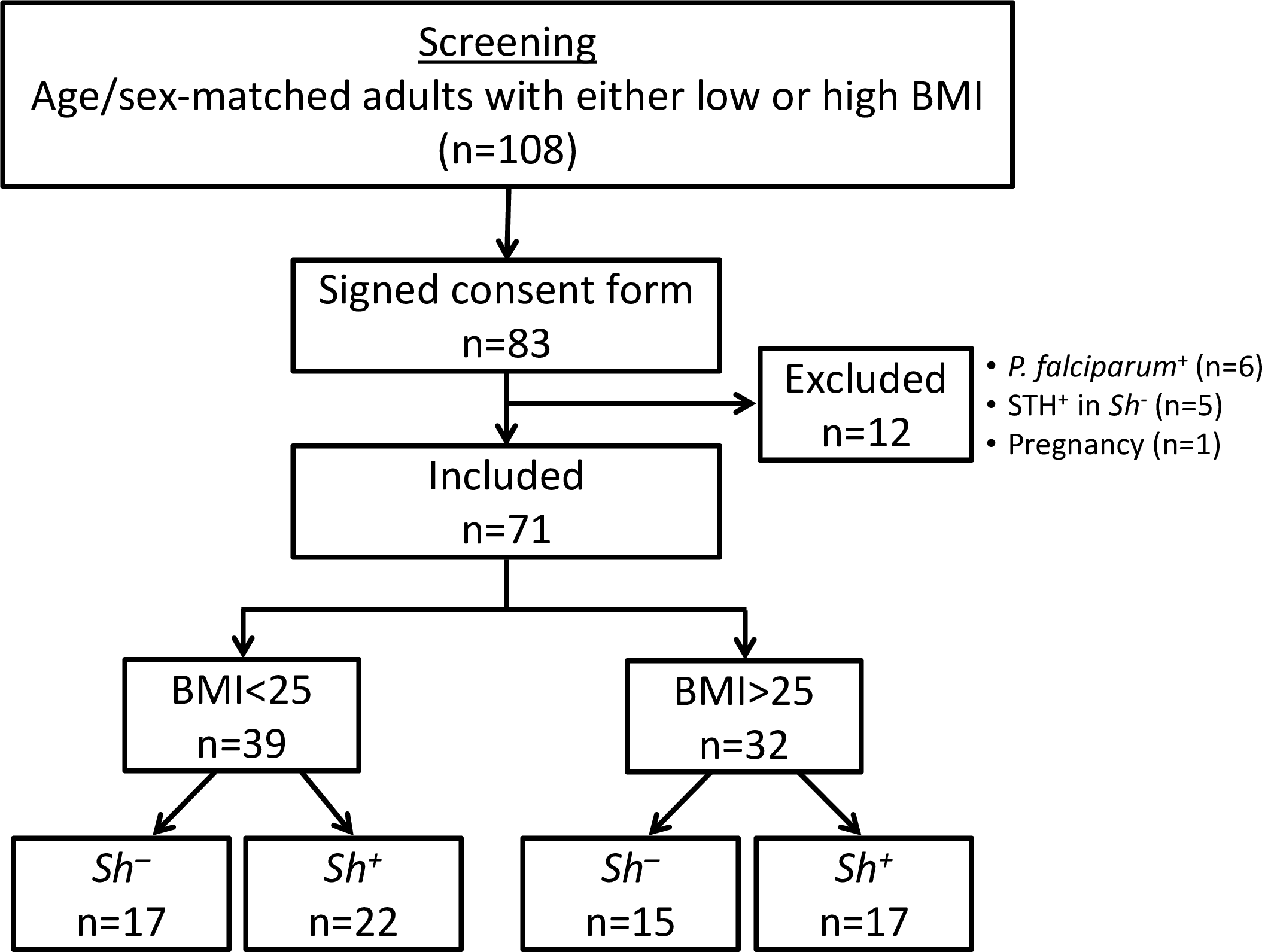

Supplement: S1 Fig — Sh, Schistosoma haematobium; STH, Soil-transmitted helminths; BMI, Body Mass Index. (TIF) [file pntd.0008464.s001.tif]

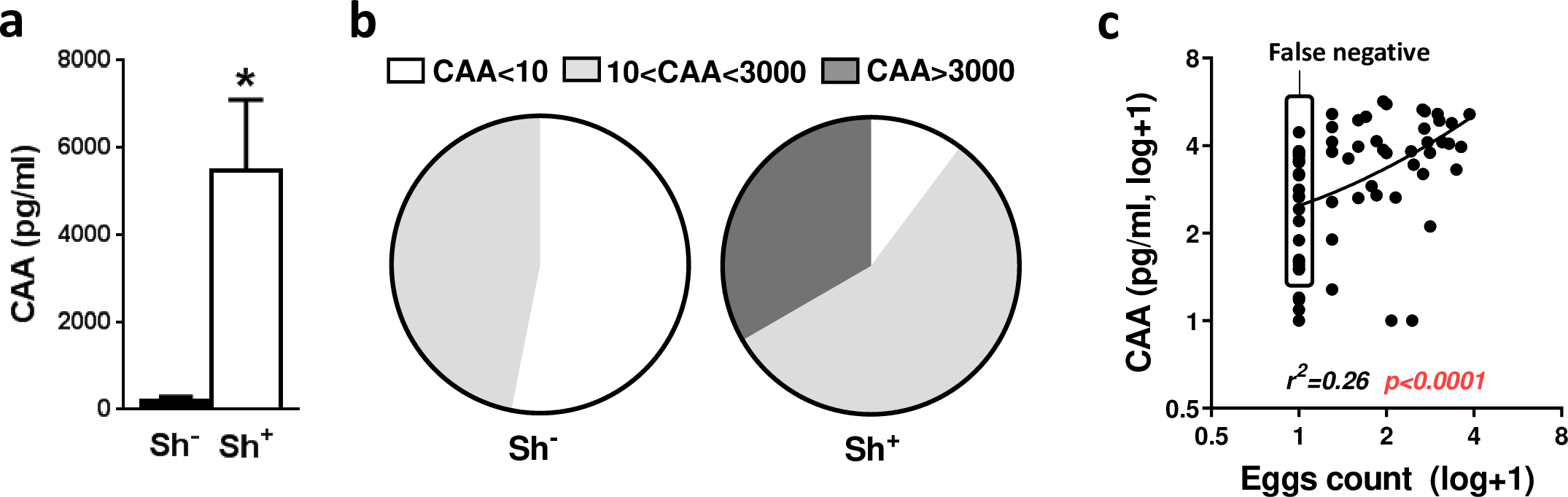

Supplement: S2 Fig — The serum CAA concentrations were measured in Sh- and Sh+ individuals diagnosed by presence of urine eggs (a), and the data were stratified in each population according to ‘below detection threshold’ (<10pg/ml), ‘medium/high’ (10<CAA<3000pg/ml) or ‘high’ (>3000pg/ml) CAA levels (b). The correlation between serum CAA levels and urine S. haematobium egg counts (c) in the whole population was plotted. (TIF) [file pntd.0008464.s002.tif]
