# Supplementary material for: Schistosoma haematobium infection is associated with lower serum cholesterol levels and improved lipid profile in overweight/obese individuals
Source: PLoS Negl Trop Dis. 2020 Jul 2;14(7):e0008464. doi: 10.1371/journal.pntd.0008464 (PMC7363109; doi:10.1371/journal.pntd.0008464)
Supplement: S1 Table — Abbreviations: CI: confidence interval; OR: Odd ratio; TIgE: total immunoglobulin E; hs-CRP: high-sensitivity C-reactive protein; ALAT: alanine aminotransferase; ASAT: aspartate aminotransferase; HOMA-IR: HOmeostatic Model Assessment for Insulin Resistance; TC: total cholesterol; HDL-C: high density lipoprotein-cholesterol; LDL-C: low density lipoprotein cholesterol; TG: triglyceride. (DOCX) [file pntd.0008464.s003.docx]

# Table S1.

|  | **Crude OR**  (95% CI) | ***P*-value** | **Adjusted OR for age, sex and BMI**  (95% CI) | ***P*-value** |
| --- | --- | --- | --- | --- |
| **TIgE** | **2.615** (1.136-6.791) | **0.032** | **2.967** (1.229-8.161) | **0.023** |
| **Eosinophils** | **1.117** (1.029-1.239) | **0.018** | **1.104** (1.016-1.226) | **0.035** |
| **hs-CRP** | **0.967** (0.874-1.037) | 0.40 | **0.966** (0.869-1.037) | 0.40 |
| **ALAT** | **0.981** (0.935-1.026) | 0.41 | **0.930** (0.930-1.027) | 0.38 |
| **ASAT** | **0.970** (0.915-1.026) | 0.29 | **0.960** (0.900-1.019) | 0.19 |
| **Glucose** | **0.670** (0.333-1.280) | 0.23 | **0.650** (0.310-1.291) | 0.23 |
| **Insulin** | **1.219** (0.765-1.956) | 0.41 | **1.018** (0.960-1.088) | 0.56 |
| **C-peptide** | **1.069** (0.397-3.111) | 0.89 | **1.156** (0.416-3.621) | 0.78 |
| **HOMA-IR** | **0.990** (0.767-1.287) | 0.94 | **1.003** (0.773-1.317) | 0.98 |
| **TC** | **0.533** (0.278-0.956) | **0.043** | **0.525** (0.251-1.010) | 0.07 |
| **HDL-C** | **0.111** (0.019-0.481) | **0.007** | **0.098** (0.016-0.450) | **0.006** |
| **LDL-C** | **0.895** (0.471-1.684) | 0.73 | **1.018** (0.501-2.097) | 0.96 |
| **TG** | **0.159** (0.022-0.717) | **0.038** | **0.115** (0.013-0.629) | **0.025** |
