# Supplementary material for: Schistosoma haematobium infection is associated with lower serum cholesterol levels and improved lipid profile in overweight/obese individuals
Source: PLoS Negl Trop Dis. 2020 Jul 2;14(7):e0008464. doi: 10.1371/journal.pntd.0008464 (PMC7363109; doi:10.1371/journal.pntd.0008464)
Supplement: S2 Table — Abbreviations: CI: confidence interval; OR: Odd ratio; TIgE: total immunoglobulin E; hs-CRP: high-sensitivity C-reactive protein; ALAT: alanine aminotransferase; ASAT: aspartate aminotransferase; HOMA-IR: HOmeostatic Model Assessment for Insulin Resistance; TC: total cholesterol; HDL-C: high density lipoprotein-cholesterol; LDL-C: low density lipoprotein cholesterol; TG: triglyceride. (DOCX) [file pntd.0008464.s004.docx]

# Table S2.

|  | **Crude OR**  (95% CI) | ***P*-value** | **Adjusted OR for age, sex and BMI**  (95% CI) | ***P*-value** |
| --- | --- | --- | --- | --- |
| **TIgE** | **3.423** (1.431-9.246) | **0.009** | **6.336** (2.217-22.53) | **0.002** |
| **Eosinophils** | **1.114** (1.018-1.252) | **0.037** | **1.112** (1.012-1.261) | 0.05 |
| **hs-CRP** | **1.131** (0.989-1.390) | 0.17 | **1.190** (1.007-1.506) | 0.09 |
| **ALAT** | **1.025** (0.974-1.094) | 0.38 | **1.027** (0.971-1.100) | 0.40 |
| **ASAT** | **1.053** (0.985-1.142) | 0.17 | **1.050** (0.975-1.149) | 0.23 |
| **Glucose** | **0.984** (0.491-2.064) | 0.96 | **1.224** (0.579-2.761) | 0.60 |
| **Insulin** | **1.027** (0.963-1.119) | 0.47 | **1.037** (0.971-1.135) | 0.33 |
| **C-peptide** | **1.085** (0.393-3.908) | 0.88 | **1.401** (0.476-5.604) | 0.57 |
| **HOMA-IR** | **1.093** (0.828-1.574) | 0.57 | **1.157** (0.866-1.069) | 0.37 |
| **TC** | **0.390** (0.181-0.757) | **0.009** | **0.427** (0.186-0.885) | **0.029** |
| **HDL-C** | **0.264** (0.057-1.053) | 0.06 | **0.261** (0.051-1.106) | 0.08 |
| **LDL-C** | **0.516** (0.239-1.031) | 0.07 | **0.592** (0.257-1.295) | 0.19 |
| **TG** | **0.432** (0.107-1.569) | 0.20 | **0.523** (0.111-2.262) | 0.38 |
