# Supplementary material for: Schistosoma haematobium infection is associated with lower serum cholesterol levels and improved lipid profile in overweight/obese individuals
Source: PLoS Negl Trop Dis. 2020 Jul 2;14(7):e0008464. doi: 10.1371/journal.pntd.0008464 (PMC7363109; doi:10.1371/journal.pntd.0008464)
Supplement: S3 Table — Normally distributed data are presented as means +/- standard deviation (SD) and non-normally distributed data as median +/- interquartile range (IQR). *, some values are missing (for TIgE n = 36 in 10<CAA<3000pg/ml; for eosinophils n = 11 in CAA<10pg/ml, n = 24 in 10<CAA<3000pg/ml and n = 10 in CAA>3000pg/ml). Abbreviations: BMI: body mass index; TIgE: total immunoglobulin E; CAA: circulating anodic antigen; hs-CRP: high-sensitivity C-reactive protein; ALAT: alanine aminotransferase; ASAT: aspartate aminotransferase; HOMA-IR: HOmeostatic Model Assessment for Insulin Resistance; TC: total cholesterol; HDL-C: high density lipoprotein-cholesterol; LDL-C: low density lipoprotein cholesterol; TG: triglyceride (DOCX) [file pntd.0008464.s005.docx]

**Table S3.**

|  | **CAA<10 pg/ml**  (n=21) | **10<CAA<3000 pg/ml**  (n=37) | **CAA>3000 pg/ml**  (n=13) | ***P*-value** |
| --- | --- | --- | --- | --- |
| **Age (year)** (mean, range) | **39.2** (18-63) | **33.7** (18-63) | **32.4** (18-57) | 0.20 |
| **Male (%)** | **42.9** | **45.9** | **53.8** |  |
| **BMI (kg/m^2^)** (mean, SD) | **27.0** (6.8) | **25.9** (5.7) | **25.2** (3.6) | 0.65 |
| **TIgE (IU/L)** (median, IQR)* | **5559** (700-17761) | **9471** (5483-17825) | **13283** (6926-22598) | **0.013** |
| **Eosinophils (%)** (mean, SD)* | **9.9** (7.5) | **15.6** (9.5) | **21.3** (9.9) | **0.024** |
| **hs-CRP (mg/L)** (median, IQR) | **1.66** (0.50-2.69) | **2.6** (0.7-6.1) | **1.6** (0.5-4.3) | 0.33 |
| **ALAT (GPT, U/L)** (mean, SD) | **16.3** (7.3) | **20.2** (12.7) | **14.7** (6.8) | 0.19 |
| **ASAT (GOT, U/L)** (mean, SD) | **23.0** (5.3) | **27.3** (10.5) | **23.0** (5.0) | 0.11 |
| **Glucose (mmol/L)** (mean, SD) | **4.44** (1.12) | **4.63** (0.78) | **4.55** (0.63) | 0.71 |
| **Insulin (mU/L)** (median, IQR) | **4.55** (2.76-7.19) | **3.91** (2.79-9.33) | **5.16** (3.19-10.06) | 0.59 |
| **C-peptide (nmol/L)** (median, IQR) | **0.09** (0.07-0.17) | **0.08** (0.05-0.15) | **0.08** (0.11-0.55) | 0.61 |
| **HOMA-IR** (median, IQR) | **1.01** (0.58-1.46) | **0.83** (0.55-1.70) | **0.99** (0.59-2.31) | 0.75 |
| **TC (mmol/L)** (mean, SD) | **4.62** (0.89) | **4.09** (0.83) | **3.79** (0.52) | **0.010** |
| **HDL-C (mmol/L)** (mean, SD) | **1.42** (0.38) | **1.26** (0.36) | **1.18** (0.38) | 0.14 |
| **LDL-C (mmol/L)** (mean, SD) | **2.78** (0.82) | **2.46** (0.75) | **2.31** (0.54) | 0.15 |
| **TG (mmol/L)** (mean, SD) | **0.91** (0.56) | **0.82** (0.28) | **0.66** (0.26) | 0.19 |
