# Supplementary material for: Schistosoma haematobium infection is associated with lower serum cholesterol levels and improved lipid profile in overweight/obese individuals
Source: PLoS Negl Trop Dis. 2020 Jul 2;14(7):e0008464. doi: 10.1371/journal.pntd.0008464 (PMC7363109; doi:10.1371/journal.pntd.0008464)
Supplement: S4 Table — Normally distributed data are presented as means +/- standard deviation (SD) and non-normally distributed data as median +/- interquartile range (IQR). Adjusted mean difference for TIgE, hs-CRP, Insulin, C-peptide and HOMA-IR were anti-log transformed. *, some values are missing (for TIgE n = 21 in BMI>25 and CAA>10pg/ml, for eosinophils n = 4 in BMI<25 and CAA<10pg/ml, n = 19 in BMI<25 and CAA>10pg/ml, n = 7 in BMI>25 and CAA<10pg/ml and n = 15 in BMI>25 and CAA>10pg/ml). Abbreviations: BMI: body mass index; TIgE: total immunoglobulin E; CAA: circulating anodic antigen; hs-CRP: high-sensitivity C-reactive protein; ALAT: alanine aminotransferase; ASAT: aspartate aminotransferase; HOMA-IR: HOmeostatic Model Assessment for Insulin Resistance; TC: total cholesterol; HDL-C: high density lipoprotein-cholesterol; LDL-C: low density lipoprotein cholesterol; TG: triglycerides. (DOCX) [file pntd.0008464.s006.docx]

# Table S4.

|  | **BMI <25** | | | | | **BMI >25** | | | | |
| --- | --- | --- | --- | --- | --- | --- | --- | --- | --- | --- |
|  | **CAA<10 pg/ml**  (n=11) | **CAA>10 pg/ml**  (n=28) | ***P*-value** | ***Mean difference adjusted for age and sex (95% CI)*** | ***P*-value** | **CAA<10 pg/ml**  (n=10) | **CAA>10 pg/ml**  (n=22) | ***P*-value** | ***Mean difference adjusted for age and sex (95% CI)*** | ***P*-value** |
| **Age (year)** (mean, range) | **39.3** (18-63) | **32.5** (18-58) | 0.93 |  |  | **39.1** (20-49) | **34.4** (18-63) | 0.49 |  |  |
| **Male (%)** | **45.5** | **46.4** |  |  |  | **40.0** | **50.0** |  |  |  |
| **BMI (kg/m^2^)** (mean, SD) | **21.9** (2.1) | **22.0** (2.1) | 0.93 | **0.3** (-1.3, 2.0) | 0.74 | **32.6** (5.6) | **30.6** (10.5) | 0.23 | **-1.0** (-4.3, 2.2) | 0.53 |
| **TIgE (IU/L)** (median, IQR)* | **3299** (596-22589) | **10470** (5377-18661) | 0.20 | **2448** (-4501, 9396) | 0.48 | **5887** (938-11859) | **10206** (6899-18669) | **0.050** | **6952** (-870, 14776) | **0.010** |
| **Eosinophils (%)** (mean, SD)* | **12.3** (8.4) | **19.3** (10.2) | 0.21 | **7.5** (-4.2, 19.3) | 0.20 | **8.5** (7.2) | **14.6** (9.0) | 0.13 | **6.6** (-1.9, 15.2) | 0.12 |
| **hs-CRP (mg/L)** (median, IQR) | **1.66** (0.54-2.32) | **1.48** (0.64-4.59) | 0.61 | **0.55** (-0.53, 1.64) | 0.31 | **1.41** (0.47-3.17) | **3.11** (0.89-7.41) | 0.19 | **0.59** (-0.37, 1.56) | 0.21 |
| **ALAT (GPT, U/L)** (mean, SD) | **15.0** (4.1) | **17.2** (9.3) | 0.45 | **2.5** (-3.8, 8.7) | 0.42 | **17.9** (9.7) | **20.7** (14.1) | 0.57 | **0.29** (-10.2, 10.8) | 0.96 |
| **ASAT (GOT, U/L)** (mean, SD) | **23.4** (5.1) | **25.1** (9.2) | 0.58 | **1.2** (-4.9, 7.2) | 0.69 | **22.6** (5.6) | **27.5** (9.9) | 0.15 | **3.9** (-3.2, 11.1) | 0.28 |
| **Glucose (mmol/L)** (mean, SD) | **4.37** (1.45) | **4.61** (0.59) | 0.46 | **0.43** (-0.22, 1.06) | 0.19 | **4.51** (0.67) | **4.60** (0.90) | 0.77 | **0.08** (-0.63, 0.79) | 0.82 |
| **Insulin (mU/L)** (median, IQR) | **4.55** (2.58-7.23) | **5.22** (2.92-10.29) | 0.43 | **0.25** (-0.46, 0.96) | 0.47 | **5.18** (2.90-7.53) | **3.93** (1.90-8.34) | 0.51 | **-0.06** (-0.92, 0.79) | 0.88 |
| **C-peptide (nmol/L)** (median, IQR) | **0.35** (0.29-0.51) | **0.43** (0.33-0.66) | 0.87 | **0.06** (-0.44, 0.58) | 0.79 | **0.49** (0.33-1.09) | **0.32** (0.24-0.60) | 0.10 | **-0.13** (-0.84, 0.22) | 0.23 |
| **HOMA-IR** (median, IQR) | **0.93** (0.38-1.20) | **1.15** (0.60-2.16) | 0.25 | **0.44** (-0.30, 1.18) | 0.23 | **1.08** (0.57-1.40) | **0.85** (0.37-1.70) | 0.55 | **-0.05** (-0.95, 0.85) | 0.91 |
| **TC (mmol/L)** (mean, SD) | **4.35** (0.89) | **3.92** (0.61) | 0.09 | **-0.35** (-0.81, 0.10) | 0.12 | **4.93** (0.82) | **4.14** (0.93) | **0.029** | **-0.50** (-1.19, 0.18) | 0.14 |
| **HDL-C (mmol/L)** (mean, SD) | **1.49** (0.48) | **1.23** (0.38) | 0.08 | **-0.26** (-0.58, 0.06) | 0.10 | **1.35** (0.23) | **1.25** (0.35) | 0.42 | **-0.04** (-0.29, 0.21) | 0.75 |
| **LDL-C (mmol/L)** (mean, SD) | **2.51** (0.83) | **2.32** (0.53) | 0.40 | **-0.13** (-0.55, 0.27) | 0.51 | **3.08** (0.73) | **2.54** (0.87) | 0.10 | **-0.33** (-1.00, 0.32) | 0.31 |
| **TG (mmol/L)** (mean, SD) | **0.75** (0.21) | **0.79** (0.25) | 0.62 | **0.09** (-0.07, 0.26) | 0.25 | **1.09** (0.76) | **0.76** (0.32) | 0.22 | **-0.30** (-0.67, 0.09) | 0.13 |
