# Supplementary material for: Schistosoma haematobium infection is associated with lower serum cholesterol levels and improved lipid profile in overweight/obese individuals
Source: PLoS Negl Trop Dis. 2020 Jul 2;14(7):e0008464. doi: 10.1371/journal.pntd.0008464 (PMC7363109; doi:10.1371/journal.pntd.0008464)
Supplement: S5 Table — Data are presented as means (+/- SD). Abbreviations: CAA: circulating anodic antigen; PC: Phosphatidylcholine; PE: Phosphatidylethanolamine; LPC: Lysophosphatidylcholine; LPE: Lysophosphatidylethanolamine; SM: Sphingomyelin; CE: Cholesterylester; DG: Diglycerides; TG: Triglycerides; FFA: Free-fatty acids. (DOCX) [file pntd.0008464.s007.docx]

**Table S5**

| Lipid species  (nmol/g) | **CAA <10**  (n=19) | | **10> CAA <3000**  (n=36) | | **CAA>3000** (n=13) | | **p-value**  <10 *vs* 10-3000 | **p-value**  <10 *vs* >3000 | **p-value**  10-3000 vs >3000 |
| --- | --- | --- | --- | --- | --- | --- | --- | --- | --- |
|  | **Mean** | SD | **Mean** | SD | **Mean** | SD |  |  |  |
| PC(16:0/16:0) | **20.8** | 3.8 | **19.2** | 3.2 | **17.3** | 2.9 | 0.98 | 0.93 | 0.98 |
| PC(16:0/16:1) | **9.6** | 6.5 | **13.8** | 13.2 | **7.8** | 3.5 | 0.85 | 0.98 | 0.78 |
| PC(16:0/18:0) | **15.2** | 3.3 | **15.0** | 2.4 | **12.9** | 2.1 | 1.00 | 0.97 | 0.97 |
| PC(16:0/18:1) | **287.3** | 89.3 | **295.6** | 102.3 | **237.1** | 50.6 | 0.54 | **<0.0001** | **<0.0001** |
| PC(16:0/18:2) | **432.0** | 95.0 | **389.8** | 78.0 | **383.8** | 87.9 | **<0.0001** | **<0.0001** | 0.78 |
| PC(16:0/18:3) | **4.8** | 2.2 | **5.4** | 2.4 | **3.5** | 1.1 | 1.00 | 0.99 | 0.97 |
| PC(16:0/20:2) | **6.0** | 1.8 | **5.6** | 1.1 | **5.0** | 0.8 | 1.00 | 0.99 | 1.00 |
| PC(16:0/20:3) | **64.9** | 21.0 | **64.3** | 17.0 | **59.4** | 14.1 | 1.00 | 0.84 | 0.85 |
| PC(16:0/20:4) | **211.3** | 63.6 | **192.5** | 52.3 | **168.4** | 36.8 | **0.042** | **<0.0001** | **0.019** |
| PC(16:0/20:5) | **36.1** | 17.4 | **23.4** | 15.8 | **15.3** | 10.0 | 0.23 | 0.09 | 0.63 |
| PC(16:0/22:4) | **7.5** | 3.4 | **7.2** | 2.6 | **6.1** | 1.9 | 1.00 | 0.99 | 0.99 |
| PC(16:0/22:5) | **41.6** | 13.6 | **31.6** | 10.2 | **24.4** | 5.7 | 0.41 | 0.19 | 0.70 |
| PC(16:0/22:6) | **161.0** | 48.3 | **106.8** | 28.5 | **92.0** | 22.0 | **<0.0001** | **<0.0001** | 0.22 |
| PC(18:0/18:1) | **35.4** | 13.9 | **34.5** | 9.7 | **26.8** | 6.2 | 0.99 | 0.66 | 0.67 |
| PC(18:0/18:2) | **165.4** | 36.1 | **149.4** | 30.5 | **138.5** | 26.2 | 0.10 | **0.018** | 0.44 |
| PC(18:0/20:3) | **26.3** | 10.1 | **24.8** | 7.5 | **22.6** | 6.5 | 0.98 | 0.93 | 0.97 |
| PC(18:0/20:4) | **100.7** | 33.6 | **86.9** | 25.2 | **76.2** | 16.6 | 0.18 | **0.036** | 0.45 |
| PC(18:0/20:5) | **20.2** | 11.3 | **11.9** | 6.4 | **7.6** | 3.8 | 0.54 | 0.41 | 0.88 |
| PC(18:0/22:5) | **12.5** | 5.7 | **9.6** | 3.7 | **7.0** | 1.6 | 0.93 | 0.84 | 0.95 |
| PC(18:0/22:6) | **50.1** | 21.9 | **32.5** | 10.1 | **26.0** | 4.7 | 0.06 | **0.040** | 0.74 |
| PC(18:1/16:1) | **4.4** | 2.2 | **5.4** | 2.7 | **4.0** | 1.3 | 0.99 | 1.00 | 0.99 |
| PC(18:1/18:1) | **11.9** | 3.5 | **12.6** | 3.6 | **10.9** | 2.6 | 0.99 | 0.99 | 0.98 |
| PC(18:1/18:2) | **38.9** | 9.9 | **37.9** | 10.0 | **37.5** | 8.5 | 0.99 | 0.99 | 1.00 |
| PC(18:1/20:3) | **4.4** | 1.7 | **4.8** | 1.5 | **4.6** | 1.2 | 1.00 | 1.00 | 1.00 |
| PC(18:1/20:4) | **16.0** | 5.2 | **15.7** | 4.3 | **14.4** | 3.6 | 1.00 | 0.99 | 0.99 |
| PC(18:1/20:5) | **2.9** | 1.3 | **2.0** | 1.1 | **1.5** | 0.9 | 0.99 | 0.99 | 1.00 |
| PC(18:1/22:6) | **5.1** | 1.4 | **4.0** | 1.1 | **3.7** | 1.1 | 0.99 | 0.99 | 1.00 |
| PC(18:2/16:1) | **3.6** | 1.2 | **3.8** | 1.3 | **3.5** | 0.9 | 1.00 | 1.00 | 1.00 |
| PC(18:2/18:2) | **7.9** | 3.1 | **7.3** | 2.8 | **8.0** | 3.1 | 1.00 | 1.00 | 1.00 |
| PC(18:2/20:4) | **4.7** | 1.3 | **4.5** | 1.4 | **4.5** | 1.2 | 1.00 | 1.00 | 1.00 |
|  |  |  |  |  |  |  |  |  |  |
| PE(16:0/18:1) | **1.6** | 0.8 | **1.6** | 0.7 | **1.1** | 0.5 | 0.99 | 0.77 | 0.67 |
| PE(16:0/18:2) | **2.0** | 1.1 | **2.1** | 0.9 | **1.6** | 0.8 | 0.98 | 0.84 | 0.70 |
| PE(16:0/20:4) | **2.6** | 1.5 | **2.9** | 1.4 | **2.0** | 0.9 | 0.82 | 0.58 | 0.23 |
| PE(16:0/22:6) | **6.8** | 3.4 | **5.3** | 2.3 | **3.8** | 1.5 | **0.008** | **<0.0001** | **0.019** |
| PE(18:0/18:1) | **4.5** | 1.6 | **4.8** | 1.6 | **3.7** | 0.7 | 0.89 | 0.37 | 0.14 |
| PE(18:0/18:2) | **7.5** | 2.6 | **7.3** | 1.9 | **6.4** | 1.9 | 0.94 | 0.16 | 0.18 |
| PE(18:0/20:3) | **1.5** | 0.7 | **1.6** | 0.5 | **1.3** | 0.4 | 0.99 | 0.94 | 0.89 |
| PE(18:0/20:4) | **9.0** | 4.5 | **8.9** | 3.1 | **6.8** | 2.3 | 0.97 | **0.001** | **0.001** |
| PE(18:0/20:5) | **1.1** | 0.8 | **0.8** | 0.7 | **0.5** | 0.2 | 0.75 | 0.55 | 0.86 |
| PE(18:0/22:5) | **1.2** | 0.6 | **1.1** | 0.5 | **0.7** | 0.2 | 0.97 | 0.72 | 0.80 |
| PE(18:0/22:6) | **7.2** | 3.9 | **5.1** | 1.8 | **3.7** | 1.0 | **<0.0001** | **<0.0001** | **0.047** |
| PE(18:1/20:4) | **1.1** | 0.5 | **1.1** | 0.4 | **0.9** | 0.3 | 1.00 | 0.97 | 0.95 |
|  |  |  |  |  |  |  |  |  |  |
| PE(O-16:0/20:4) | **1.2** | 0.4 | **1.1** | 0.6 | **1.1** | 0.3 | 0.52 | 0.70 | 1.00 |
| PE(O-16:0/22:6) | **1.8** | 0.7 | **1.4** | 0.5 | **1.2** | 0.3 | **0.001** | **0.002** | 0.74 |
| PE(O-18:0/20:4) | **1.4** | 0.5 | **1.3** | 0.5 | **1.2** | 0.2 | 0.79 | 0.41 | 0.68 |
| PE(O-18:0/22:6) | **1.3** | 0.5 | **1.0** | 0.3 | **0.9** | 0.2 | **0.037** | **0.025** | 0.74 |
|  |  |  |  |  |  |  |  |  |  |
| PE(P-16:0/18:1) | **1.3** | 0.4 | **1.4** | 0.6 | **1.2** | 0.4 | 0.98 | 0.99 | 0.94 |
| PE(P-16:0/18:2) | **2.7** | 1.0 | **3.0** | 1.6 | **2.5** | 1.0 | 0.80 | 0.98 | 0.73 |
| PE(P-16:0/20:4) | **10.4** | 3.9 | **10.5** | 5.8 | **8.2** | 2.5 | 0.98 | **0.014** | **0.003** |
| PE(P-16:0/20:5) | **1.7** | 1.0 | **1.2** | 0.7 | **0.8** | 0.4 | 0.61 | 0.41 | 0.83 |
| PE(P-16:0/22:5) | **2.7** | 0.8 | **2.8** | 1.1 | **2.0** | 0.6 | 1.00 | 0.64 | 0.54 |
| PE(P-16:0/22:6) | **9.8** | 3.3 | **7.6** | 2.6 | **6.1** | 1.3 | **0.001** | **<0.0001** | 0.08 |
| PE(P-18:0/18:1) | **1.1** | 0.3 | **1.2** | 0.5 | **0.9** | 0.2 | 0.98 | 0.98 | 0.92 |
| PE(P-18:0/18:2) | **3.3** | 1.2 | **3.6** | 1.5 | **3.1** | 1.0 | 0.85 | 0.95 | 0.70 |
| PE(P-18:0/20:3) | **1.0** | 0.4 | **1.2** | 0.5 | **0.9** | 0.2 | 0.98 | 0.99 | 0.95 |
| PE(P-18:0/20:4) | **14.7** | 5.4 | **13.9** | 5.3 | **11.4** | 2.6 | 0.41 | **<0.0001** | **0.001** |
| PE(P-18:0/20:5) | **3.8** | 2.2 | **2.3** | 1.3 | **1.5** | 0.7 | **0.042** | **0.009** | 0.48 |
| PE(P-18:0/22:5) | **1.4** | 0.4 | **1.4** | 0.6 | **1.0** | 0.2 | 1.00 | 0.86 | 0.81 |
| PE(P-18:0/22:6) | **9.6** | 3.6 | **7.4** | 2.3 | **5.9** | 1.1 | **0.000** | **<0.0001** | 0.10 |
| PE(P-18:1/18:2) | **1.2** | 0.5 | **1.3** | 0.6 | **1.1** | 0.4 | 0.97 | 0.99 | 0.94 |
| PE(P-18:1/20:4) | **5.7** | 2.2 | **5.6** | 2.1 | **4.6** | 1.2 | 0.98 | 0.26 | 0.25 |
| PE(P-18:1/20:5) | **1.2** | 0.8 | **0.7** | 0.4 | **0.5** | 0.2 | 0.71 | 0.63 | 0.94 |
| PE(P-18:1/22:6) | **4.5** | 1.8 | **3.4** | 1.0 | **2.7** | 0.6 | 0.16 | 0.06 | 0.60 |
|  |  |  |  |  |  |  |  |  |  |
| LPC(16:0) | **128.5** | 43.6 | **104.0** | 25.5 | **98.6** | 24.7 | **<0.0001** | **<0.0001** | 0.36 |
| LPC(16:1) | **1.2** | 0.6 | **1.3** | 0.7 | **1.1** | 0.4 | 1.00 | 1.00 | 1.00 |
| LPC(17:0) | **1.7** | 1.4 | **1.1** | 0.5 | **1.0** | 0.3 | 0.99 | 0.99 | 1.00 |
| LPC(18:0) | **42.1** | 19.1 | **31.8** | 9.2 | **29.8** | 7.9 | **0.009** | **0.016** | 0.88 |
| LPC(18:1) | **23.3** | 6.6 | **22.4** | 7.2 | **19.9** | 5.6 | 0.96 | 0.72 | 0.81 |
| LPC(18:2) | **12.0** | 3.6 | **11.1** | 4.1 | **10.9** | 4.5 | 0.96 | 0.97 | 1.00 |
| LPC(20:3) | **1.9** | 0.7 | **1.9** | 0.7 | **1.8** | 0.6 | 1.00 | 1.00 | 1.00 |
| LPC(20:4) | **3.3** | 1.1 | **2.9** | 0.9 | **2.8** | 0.8 | 0.99 | 0.99 | 1.00 |
|  |  |  |  |  |  |  |  |  |  |
| LPE(16:0) | **1.2** | 0.5 | **1.1** | 0.4 | **0.9** | 0.3 | 0.45 | 0.16 | 0.59 |
| LPE(18:0) | **1.5** | 0.8 | **1.2** | 0.4 | **1.1** | 0.3 | 0.10 | **0.021** | 0.46 |
|  |  |  |  |  |  |  |  |  |  |
| SM(14:0) | **10.6** | 6.1 | **8.1** | 3.7 | **5.9** | 2.1 | 0.98 | 0.95 | 0.99 |
| SM(16:0) | **326.4** | 138.3 | **287.8** | 107.9 | **244.9** | 82.6 | **0.003** | **<0.0001** | **0.004** |
| SM(18:0) | **57.6** | 25.0 | **48.5** | 20.2 | **43.1** | 14.8 | 0.72 | 0.60 | 0.92 |
| SM(18:1) | **10.5** | 3.1 | **8.7** | 2.6 | **9.0** | 2.6 | 0.99 | 0.99 | 1.00 |
| SM(20:0) | **219.3** | 80.5 | **209.9** | 70.3 | **194.0** | 83.1 | 0.71 | 0.21 | 0.47 |
| SM(20:1) | **7.7** | 1.9 | **7.6** | 1.9 | **7.2** | 1.8 | 1.00 | 1.00 | 1.00 |
| SM(22:0) | **132.6** | 40.6 | **127.3** | 38.7 | **109.5** | 39.8 | 0.90 | 0.28 | 0.39 |
| SM(22:1) | **45.4** | 9.3 | **46.2** | 9.2 | **43.6** | 7.7 | 1.00 | 0.99 | 0.98 |
| SM(24:0) | **54.4** | 12.5 | **53.3** | 12.3 | **47.7** | 14.1 | 1.00 | 0.89 | 0.91 |
| SM(24:1) | **61.0** | 11.4 | **58.6** | 10.5 | **56.4** | 8.6 | 0.98 | 0.95 | 0.99 |
| SM(26:0) | **1.8** | 0.5 | **1.7** | 0.4 | **2.0** | 1.0 | 1.00 | 1.00 | 1.00 |
| SM(26:1) | **3.2** | 0.5 | **3.3** | 0.6 | **3.2** | 0.4 | 1.00 | 1.00 | 1.00 |
|  |  |  |  |  |  |  |  |  |  |
| CE(14:0) | **45.7** | 48.5 | **31.8** | 31.6 | **26.5** | 20.8 | 0.84 | 0.81 | 0.98 |
| CE(15:0) | **10.1** | 9.0 | **7.7** | 7.3 | **6.7** | 6.5 | 0.99 | 0.99 | 1.00 |
| CE(16:0) | **421.7** | 103.3 | **348.0** | 86.5 | **306.9** | 50.9 | **0.008** | **0.001** | 0.30 |
| CE(16:1) | **95.3** | 46.8 | **97.6** | 62.9 | **75.4** | 27.6 | 1.00 | 0.80 | 0.71 |
| CE(17:0) | **12.6** | 10.1 | **12.0** | 13.0 | **9.1** | 6.9 | 1.00 | 0.99 | 0.99 |
| CE(18:0) | **71.5** | 47.9 | **61.8** | 49.1 | **55.1** | 34.1 | 0.92 | 0.86 | 0.97 |
| CE(18:1) | **659.1** | 165.8 | **594.8** | 167.5 | **523.1** | 94.6 | **0.024** | **<0.0001** | **0.028** |
| CE(18:2) | **1477.6** | 273.1 | **1203.0** | 301.6 | **1132.3** | 219.3 | **<0.0001** | **<0.0001** | **0.031** |
| CE(18:3) | **48.8** | 19.6 | **42.7** | 14.3 | **34.0** | 8.9 | 0.97 | 0.88 | 0.95 |
| CE(18:4) | **2.3** | 1.6 | **1.9** | 1.8 | **1.0** | 0.4 | 1.00 | 1.00 | 1.00 |
| CE(20:3) | **26.0** | 9.6 | **21.9** | 7.5 | **20.4** | 5.9 | 0.98 | 0.98 | 1.00 |
| CE(20:4) | **312.9** | 117.6 | **249.5** | 82.6 | **221.5** | 72.0 | **0.026** | **0.009** | 0.58 |
| CE(20:5) | **79.4** | 41.4 | **41.7** | 25.1 | **27.9** | 9.9 | 0.27 | 0.22 | 0.87 |
| CE(22:0) | **6.3** | 6.3 | **3.3** | 4.2 | **2.0** | 1.5 | 0.99 | 0.99 | 1.00 |
| CE(22:5) | **5.7** | 2.7 | **3.8** | 1.4 | **3.2** | 1.0 | 1.00 | 1.00 | 1.00 |
| CE(22:6) | **51.4** | 20.5 | **30.8** | 11.0 | **26.2** | 9.0 | 0.68 | 0.70 | 0.99 |
| CE(24:0) | **4.2** | 4.4 | **2.3** | 2.2 | **4.8** | 6.5 | 1.00 | 1.00 | 1.00 |
|  |  |  |  |  |  |  |  |  |  |
| DG(16:0/18:1) | **3.6** | 3.0 | **3.0** | 1.3 | **2.3** | 1.2 | 0.13 | **0.007** | 0.20 |
| DG(16:0/18:2) | **1.2** | 0.8 | **0.9** | 0.4 | **0.8** | 0.4 | 0.60 | 0.63 | 0.98 |
| DG(18:1/18:1) | **3.1** | 1.6 | **2.9** | 1.1 | **2.5** | 1.1 | 0.80 | 0.33 | 0.56 |
| DG(18:1/18:2) | **2.3** | 1.0 | **1.9** | 0.6 | **1.8** | 0.7 | 0.42 | 0.54 | 1.00 |
|  |  |  |  |  |  |  |  |  |  |
| TG44:0 | **2.1** | 4.8 | **1.7** | 3.3 | **0.9** | 1.5 | 1.00 | 1.00 | 1.00 |
| TG44:1 | **1.8** | 4.1 | **1.5** | 2.3 | **0.8** | 1.2 | 1.00 | 1.00 | 1.00 |
| TG46:0 | **6.1** | 12.6 | **5.1** | 9.0 | **2.4** | 3.2 | 1.00 | 0.96 | 0.97 |
| TG46:1 | **9.9** | 20.0 | **8.1** | 11.2 | **4.4** | 5.9 | 0.98 | 0.92 | 0.95 |
| TG46:2 | **3.9** | 6.6 | **3.8** | 4.5 | **2.3** | 2.8 | 1.00 | 0.99 | 0.99 |
| TG47:0 | **1.3** | 2.7 | **0.9** | 1.4 | **0.4** | 0.4 | 1.00 | 1.00 | 1.00 |
| TG47:1 | **1.4** | 2.9 | **0.8** | 1.1 | **0.4** | 0.3 | 1.00 | 1.00 | 1.00 |
| TG48:0 | **18.9** | 31.2 | **17.6** | 20.6 | **8.6** | 10.3 | 0.99 | 0.74 | 0.75 |
| TG48:1 | **46.3** | 75.6 | **35.8** | 37.9 | **20.7** | 21.7 | 0.60 | 0.15 | 0.44 |
| TG48:2 | **27.4** | 38.6 | **22.7** | 19.7 | **14.9** | 13.9 | 0.91 | 0.64 | 0.81 |
| TG48:3 | **7.0** | 8.8 | **6.2** | 5.2 | **4.2** | 3.8 | 1.00 | 0.98 | 0.99 |
| TG48:4 | **1.3** | 1.9 | **1.3** | 1.4 | **0.8** | 0.8 | 1.00 | 1.00 | 1.00 |
| TG49:0 | **1.7** | 3.4 | **1.3** | 1.9 | **0.6** | 0.5 | 1.00 | 1.00 | 1.00 |
| TG49:1 | **6.6** | 11.2 | **4.4** | 3.9 | **2.7** | 1.7 | 0.98 | 0.96 | 0.99 |
| TG49:2 | **3.9** | 5.7 | **2.8** | 2.1 | **1.9** | 1.1 | 0.99 | 0.99 | 1.00 |
| TG50:0 | **19.2** | 25.3 | **16.9** | 14.5 | **9.3** | 8.8 | 0.98 | 0.76 | 0.82 |
| TG50:1 | **179.8** | 187.4 | **141.3** | 84.4 | **96.9** | 83.6 | **0.001** | **<0.0001** | **0.001** |
| TG50:2 | **169.2** | 159.9 | **136.6** | 71.3 | **102.0** | 60.9 | **0.008** | **<0.0001** | **0.015** |
| TG50:3 | **59.2** | 52.5 | **45.8** | 23.1 | **37.5** | 19.6 | 0.44 | 0.26 | 0.78 |
| TG50:4 | **12.0** | 12.6 | **8.5** | 4.9 | **6.6** | 4.2 | 0.95 | 0.92 | 0.99 |
| TG50:5 | **2.1** | 3.1 | **1.3** | 1.0 | **0.8** | 0.5 | 1.00 | 1.00 | 1.00 |
| TG51:0 | **1.0** | 1.8 | **0.8** | 1.1 | **0.5** | 0.4 | 1.00 | 1.00 | 1.00 |
| TG51:1 | **7.0** | 10.2 | **5.3** | 4.7 | **3.3** | 2.3 | 0.99 | 0.96 | 0.99 |
| TG51:2 | **10.8** | 12.0 | **8.5** | 4.8 | **6.2** | 2.9 | 0.98 | 0.94 | 0.98 |
| TG51:3 | **3.2** | 2.9 | **2.4** | 1.3 | **2.0** | 1.0 | 1.00 | 1.00 | 1.00 |
| TG51:4 | **1.3** | 1.3 | **0.9** | 0.5 | **0.7** | 0.4 | 1.00 | 1.00 | 1.00 |
| TG52:0 | **5.5** | 6.5 | **5.3** | 4.0 | **2.9** | 2.0 | 1.00 | 0.98 | 0.98 |
| TG52:1 | **83.7** | 71.5 | **73.4** | 34.7 | **51.8** | 31.9 | 0.61 | 0.05 | 0.19 |
| TG52:2 | **448.8** | 254.5 | **384.2** | 135.8 | **323.4** | 161.0 | **<0.0001** | **<0.0001** | **<0.0001** |
| TG52:3 | **373.0** | 182.4 | **294.7** | 100.4 | **276.6** | 131.6 | **<0.0001** | **<0.0001** | 0.32 |
| TG52:4 | **125.0** | 73.3 | **90.0** | 31.0 | **84.0** | 36.9 | **0.004** | **0.009** | 0.88 |
| TG52:5 | **23.9** | 23.3 | **15.3** | 6.7 | **11.7** | 5.2 | 0.71 | 0.65 | 0.95 |
| TG52:6 | **5.2** | 7.8 | **2.8** | 2.1 | **1.6** | 0.8 | 0.97 | 0.96 | 1.00 |
| TG52:7 | **1.4** | 2.3 | **0.9** | 0.9 | **0.5** | 0.5 | 1.00 | 1.00 | 1.00 |
| TG53:0 | **1.3** | 1.8 | **0.6** | 0.5 | **0.4** | 0.2 | 1.00 | 1.00 | 1.00 |
| TG53:1 | **2.1** | 2.7 | **1.8** | 1.7 | **1.2** | 1.1 | 1.00 | 1.00 | 1.00 |
| TG53:2 | **6.6** | 5.9 | **6.0** | 3.6 | **4.4** | 2.5 | 1.00 | 0.99 | 0.99 |
| TG53:3 | **3.3** | 2.4 | **2.7** | 1.5 | **2.3** | 1.3 | 1.00 | 1.00 | 1.00 |
| TG53:4 | **2.3** | 1.8 | **1.8** | 0.8 | **1.5** | 0.6 | 1.00 | 1.00 | 1.00 |
| TG54:1 | **8.0** | 7.0 | **7.9** | 4.3 | **5.2** | 3.5 | 1.00 | 0.98 | 0.97 |
| TG54:2 | **42.2** | 26.7 | **39.9** | 16.2 | **28.6** | 17.5 | 0.98 | 0.59 | 0.64 |
| TG54:3 | **105.7** | 52.6 | **98.1** | 35.8 | **76.9** | 40.6 | 0.77 | 0.09 | 0.20 |
| TG54:4 | **93.0** | 48.4 | **78.1** | 27.5 | **64.0** | 32.6 | 0.36 | 0.09 | 0.50 |
| TG54:5 | **61.1** | 44.7 | **44.9** | 16.5 | **35.1** | 18.8 | 0.30 | 0.15 | 0.71 |
| TG54:6 | **35.1** | 37.5 | **20.6** | 9.6 | **14.5** | 7.3 | 0.38 | 0.30 | 0.88 |
| TG54:7 | **10.8** | 13.3 | **5.7** | 3.4 | **3.4** | 1.5 | 0.89 | 0.86 | 0.98 |
| TG54:8 | **1.6** | 2.2 | **0.8** | 0.5 | **0.5** | 0.3 | 1.00 | 1.00 | 1.00 |
| TG55:1 | **1.9** | 1.7 | **1.0** | 0.5 | **0.7** | 0.3 | 1.00 | 1.00 | 1.00 |
| TG56:3 | **3.4** | 2.5 | **3.3** | 2.1 | **2.3** | 1.2 | 1.00 | 1.00 | 1.00 |
| TG56:4 | **5.7** | 3.6 | **5.3** | 2.7 | **3.7** | 1.9 | 1.00 | 0.99 | 0.99 |
| TG56:5 | **16.3** | 10.1 | **13.7** | 5.8 | **10.1** | 5.9 | 0.97 | 0.90 | 0.95 |
| TG56:6 | **31.1** | 23.5 | **21.9** | 8.9 | **16.0** | 8.4 | 0.67 | 0.52 | 0.88 |
| TG56:7 | **38.4** | 34.2 | **23.2** | 11.3 | **16.1** | 7.3 | 0.34 | 0.24 | 0.84 |
| TG56:8 | **17.7** | 16.8 | **9.4** | 4.9 | **6.8** | 3.1 | 0.73 | 0.71 | 0.98 |
| TG58:6 | **2.4** | 1.8 | **2.0** | 1.2 | **1.3** | 0.8 | 1.00 | 1.00 | 1.00 |
| TG58:7 | **5.8** | 4.5 | **4.3** | 2.4 | **2.8** | 1.5 | 0.99 | 0.97 | 0.99 |
| TG58:8 | **8.0** | 6.3 | **5.3** | 2.7 | **3.6** | 1.8 | 0.97 | 0.95 | 0.99 |
| TG58:9 | **6.2** | 6.0 | **3.5** | 1.8 | **2.5** | 1.2 | 0.97 | 0.96 | 1.00 |
| TG58:10 | **4.1** | 5.9 | **1.7** | 1.1 | **1.1** | 0.5 | 0.97 | 0.97 | 1.00 |
| TG60:11 | **1.4** | 1.9 | **0.7** | 0.6 | **0.3** | 0.2 | 1.00 | 1.00 | 1.00 |
| TG60:12 | **1.3** | 1.7 | **0.5** | 0.6 | **0.2** | 0.1 | 1.00 | 1.00 | 1.00 |
|  |  |  |  |  |  |  |  |  |  |
| FFA(12:0) | **1.5** | 0.7 | **1.5** | 0.6 | **1.6** | 0.7 | 1.00 | 1.00 | 0.99 |
| FFA(14:0) | **6.4** | 2.8 | **6.0** | 3.3 | **6.9** | 4.9 | 1.00 | 1.00 | 1.00 |
| FFA(15:0) | **1.4** | 0.4 | **1.3** | 0.4 | **1.4** | 0.6 | 0.09 | 0.99 | 0.12 |
| FFA(16:0) | **137.3** | 54.6 | **121.8** | 66.2 | **138.5** | 93.5 | 1.00 | 0.94 | 0.91 |
| FFA(16:1) | **13.0** | 7.5 | **12.7** | 9.6 | **16.2** | 13.4 | 1.00 | 1.00 | 1.00 |
| FFA(17:0) | **3.0** | 0.7 | **2.8** | 0.9 | **2.9** | 1.2 | 1.00 | 1.00 | 0.99 |
| FFA(18:0) | **46.3** | 13.7 | **46.8** | 25.3 | **45.8** | 26.2 | 0.13 | 0.68 | **0.023** |
| FFA(18:1) | **145.6** | 69.9 | **131.2** | 81.5 | **153.6** | 111.8 | 0.39 | 0.94 | 0.28 |
| FFA(18:2) | **54.5** | 23.5 | **44.8** | 25.6 | **57.7** | 41.5 | 1.00 | 1.00 | 1.00 |
| FFA(18:3) | **3.1** | 1.0 | **2.8** | 1.9 | **3.2** | 2.4 | 1.00 | 1.00 | 1.00 |
| FFA(20:0) | **1.7** | 0.5 | **1.8** | 0.5 | **1.5** | 0.6 | 1.00 | 1.00 | 1.00 |
| FFA(20:1) | **1.6** | 0.7 | **1.5** | 0.9 | **1.6** | 1.0 | 1.00 | 1.00 | 1.00 |
| FFA(20:2) | **1.1** | 0.4 | **1.0** | 0.5 | **1.1** | 0.6 | 1.00 | 1.00 | 1.00 |
| FFA(20:3) | **1.6** | 0.4 | **1.6** | 0.6 | **1.7** | 0.8 | 1.00 | 1.00 | 1.00 |
| FFA(20:4) | **8.5** | 2.6 | **7.8** | 3.2 | **8.1** | 2.5 | 1.00 | 1.00 | 1.00 |
| FFA(20:5) | **1.6** | 0.8 | **1.1** | 0.6 | **0.9** | 0.3 | 0.85 | 0.93 | 0.66 |
| FFA(22:0) | **22.9** | 15.0 | **27.0** | 17.1 | **19.5** | 17.2 | 1.00 | 1.00 | 1.00 |
| FFA(22:1) | **1.8** | 0.8 | **1.5** | 0.7 | **1.7** | 1.2 | 1.00 | 1.00 | 1.00 |
| FFA(22:5) | **2.4** | 0.9 | **2.1** | 1.1 | **1.9** | 1.0 | 0.98 | 0.98 | 1.00 |
| FFA(22:6) | **6.8** | 2.6 | **5.3** | 2.8 | **4.8** | 2.1 | 1.00 | 1.00 | 0.99 |
