# Supplementary material for: Schistosoma haematobium infection is associated with lower serum cholesterol levels and improved lipid profile in overweight/obese individuals
Source: PLoS Negl Trop Dis. 2020 Jul 2;14(7):e0008464. doi: 10.1371/journal.pntd.0008464 (PMC7363109; doi:10.1371/journal.pntd.0008464)
Supplement: Attachment — Submitted filename: Points_to_points_reply_to_reviewers_PNTD-D-20-00359_R1.pdf [file pntd.0008464.s008.pdf]

Associate Editor

This study provides insight into the timely topic of helminth induced modulation of host metabolism. The reviewers are overall enthusiastic about the manuscript, but have a few minor issues that need to be addressed for acceptance. Please address the points raised by Reviewers 2 and 3 on sample size, co-infections, the use of CAA, and expanding the discussion to align the current study with the recent literature. As well as addressing the mislabeling raised by reviewers 1 and 3.

Authors' reply:

We thank the Associate Editor and the reviewers for their positive evaluation and helpful comments that have allowed us to improve our manuscript

Reviewer's Responses to Questions

Methods

Reviewer #1:

Methods are appropriate and the study design addresses the stated objectives. Although the population size is small

Reviewer #2:

The authors have performed a well-designed, reasonably well powered and thorough analysis of metabolic parameters in each cohort. The statistical analysis is appropriate and of excellent standard. The study is novel in regards to the depth of analysis, and the human population (ie concurrent analysis of obese and lean individuals +/- worms. One confounder is that is addition to S. hematobium (Sh), 7 of the participants also had another helminth infection. This is acknowledged by the authors, but it means that within their Sh+ cohort, there are people with Sh only, and some people with Sh plus other parasites. This may have implications for their results, and the authors should present some data whether the presence of a co-infection leads to quantitative differences in any of the key parameters.

Authors' reply:

We have indeed deliberately kept the Sh+ individuals infected with other helminths within our population cohort, notably because it is well established that individuals infected with one helminth specie have an increased probability to be infected with additional ones. We have therefore systematically add this parameter in our multivariate linear regression model used for adjustment for confounding factors. Altogether, the impact of infection with other helminths is negligible and removing these 7 subjects from our analysis only slightly reduces the statistical power but does not affect at all our conclusions (see Table 1 for Reviewer below; similar results observed for the other Tables/Figures [*data not shown]*). Since we already have quite a lot of Supplementary Tables we will not show this new analyses but instead we have added a sentence in the result section emphasizing that removing Sh+ individuals with other helminth infection has only marginal impact on the results and does not affect our conclusions (line 188-190, page 11).

Table 1 for reviewers. Characteristics of the study population without other helminth infection

	S. haematobium negative (n=32)	*S. haematobium* positive (n=32)	*P*-value	Mean difference adjusted for age, sex, BMI and other helminths (95% CI)	*P*-value
**Age (year)** (mean, range)	**35.7** (18-63)	**35.8** (18-63)	0.98		
**Male (%)**	**43.8**	**50**			
**BMI (kg/m^2)** (mean, SD)	**26.8** (6.9)	**25.3** (4.3)	0.31		
***S. haematobium* urine eggs** (median,	**0** (0-0)	**13** (4-66)			
**Other helminths (%)**	**0**	**0**			
***Ascaris lumbricoides* (%)**	**0**	**0**			
***Trichuris trichiura* (%)**	**0**	**0**			
***Necator americanus* (%)**	**0**	**0**			
Strongyloides (%)	**0**	**0**			
**TIgE (IU/L)** (median, IQR)*	**6216** (1545-	**9116** (6364-9116)	**0.057**	**3168** (-1715, 8053)	0.20
**Eosinophils (%)** (mean, SD)*	**10.3** (8.2)	**18.2** (10.01)	**0.013**	**7.7** (1.0, 14.5)	**0.026**
**hs-CRP (mg/L)** (median, IQR)	**1.91** (0.49-4.52)	**1.63** (0.71-4.06)	0.75	**-0.11** (-0.80, 0.57)	0.74
**ALAT (GPT, U/L)** (mean, SD)	**19.2** (11.9)	**16.0** (7.5)	0.21	**-3.3** (-8.2, 1.6)	0.18
**ASAT (GOT, U/L)** (mean, SD)	**26.4** (10.1)	**23.8** (6.5)	0.23	**-2.9** (-7.0, 1.1)	0.15
**Glucose (mmol/L)** (mean, SD)	**4.61** (1.06)	**4.46** (0.61)	0.51	**-0.17** (-0.59, 0.25)	0.43
**Insulin (mU/L)** (median, IQR)	**4.45** (2.74-7.21)	**4.44** (2.92-9.45)	0.84	**0.09** (-0.41, 0.59)	0.71
**C-peptide (nmol/L)** (median, IQR)	**0.41** (0.32-0.61)	**0.36** (0.27-0.36)	0.77	**-0.02** (-0.34, 0.30)	0.90
**HOMA-IR** (median, IQR)	**0.94** (0.52-1.32)	**0.95** (0.58-1.74)	0.84	**0.09** (-0.43, 0.61)	0.72
**TC (mmol/L)** (mean, SD)	**4.42** (0.84)	**4.07** (0.78)	**0.076**	**-0.28** (-0.66, -0.10)	0.14
**HDL-C (mmol/L)** (mean, SD)	**1.44** (0.40)	**1.19** (0.31)	**0.008**	**-0.25** (-0.43, -0.06)	**0.009**
**LDL-C (mmol/L)** (mean, SD)	**2.56** (0.73)	**2.54** (0.76)	0.93	**-0.05** (-0.30, 0.41)	0.76
**TG (mmol/L)** (mean, SD)	**0.93** (0.50)	**0.74** (0.22)	**0.048**	**-0.19** (-0.37, -0.01)	**0.037**

Reviewer #3:

Study population:

The authors do not mention how the sample size was calculated. Please include this information in the Methods section. One of the main limitations of this study is the sample size. This information is crucial to determine if the sample size is appropriate, and therefore the conclusions can be supported.

Authors' reply:

We agree with the reviewer that one of the limitation of our study, as also underlined in the discussion section (page 15), is its rather small sample size. To roughly determine the sample size, we used the average value for total cholesterol levels in a previous small cohort study performed in Lambaréné. For this primary outcome, we aimed to be able to detect a mean difference of ~12,5% between Sh- and Sh+ group, with alpha = 0.05 and a power of 80%. The number of volunteers to be recruited was calculated to be 33 per group. Taking into account the infection prevalence in the study area, a compliance rate of ~80% at screening, and a 5-10% drop-out rate after inclusion (*e.g. P. falciparum* infection), ~110 individuals were intended to be screened, among which 71 were finally included. This information has been added to the Methods section (line 97-99, Page 7).

Individuals Sh+ were negative for STH and Plasmodium? It is not clear in the Methods if co-infections were excluded.

Authors' reply:

All the individuals found to be positive for *Plasmodium falciparum,* in both Sh- and Sh+ groups, were excluded (see Figure S1). We agree that this was not crystal clear in the method section, so

we have adjusted the sentence accordingly (line 108-111, Page 7). Concerning the co-infection with STH, we have deliberately decided not to exclude the 7 Sh+ individuals found to be infected with other helminths (see response to Reviewer 2 above). As underlined, the impact of infection with other helminths is negligible and removing them from our analyses only reduces the statistical power but does not affect our conclusions.

Line 112: for treatment of Sh+ individuals, parasitological (presence of eggs in urine) and CAA results were considered?

Authors' reply:
For antihelminthic treatment of Sh+ individuals, the presence of urine eggs was used as readout for infection (CAA detection was not done during the field study but months later in the whole samples collection, together with other serum parameters). We have now added this information (line 115-116, page 8).

I strongly recommend including the reference values of the biochemical parameters for your study population as supplemental material.

Authors' reply:
Reference values for biochemical parameters at the whole population level are usually well-established in Westernized countries but are not always easily available for African individuals. We provided to the reviewer (see Table 2 for reviewers below) some of the available information obtained from local/national Gabonese health care system but we think it will not be of crucial interest to add them as supplementary data. Of note, the average values for all the biochemical parameters were within the 'normal' physiological ranges for the different groups.

Table 2 for reviewers. Reference values for biochemical parameters in Gabonese individuals

	Reference values (healthy adult - fasted)
**TIgE (IU/L)***	Not defined
**Eosinophils (%)***	Not defined
**hs-CRP (mg/L)***	Not defined
**ALAT (GPT, U/L)***	Not defined
**ASAT (GOT, U/L)***	Not defined
**Glucose (mmol/L)***	**4.11-5.55**
**Insulin (mU/L)[$]**	**3-32**
**C-peptide (nmol/L)[&]**	**0.3-0.6**
**HOMA-IR**	Not defined[a]
**TC (mmol/L)[#]**	**3.60-6.80**
**HDL-C (mmol/L)[#]**	**0.90-1.50**
**LDL-C (mmol/L)[#]**	**2.27-4.14**
**TG (mmol/L)[#]**	**0.60-1.88**

*, CERMEL, Lambarene, Gabon
#, National Public Health, Gabon
$, Chevenne et al. Diabetes Metab. 1999
&, Leighton et al. Diabetes Ther. 2017
[a], 0.5-1.4 in healthy Caucasian adults

Quantitative insulin sensitivity check index (QUICKI) = 1 / (log(fasting insulin µU/mL) + log(fasting glucose mg/dL)) should be calculated and included as an additional measurement of insulin resistance.

Authors' reply:

We agree that calculating the QUICKI index might be an alternative to HOMA-IR for assessing whole-body insulin resistance. However, we do not find any differences using both methods, whatever the conditions (see Table 3 for reviewers as example below). Taking into account that HOMA-IR is by far the most common index used and that it has also been previously used in the few publications investigating the impact of helminths on metabolic homeostasis in humans, we decided not to include this redundant calculated parameter in our already large tables.

Table 1 for reviewers. Characteristics of the study population (with QUICKI index added)

	S. haematobium negative (n=32)	*S. haematobium* positive (n=39)	*P*-value	Mean difference adjusted for age, sex, BMI and other helminths (95% CI)	*P*-value
**Age (year)** (mean, range)	**35.7** (18-63)	**34.5** (18-63)	0.68		
**Male (%)**	**43.8**	**48.7**			
**BMI (kg/m²)** (mean, SD)	**26.8** (6.9)	**25.6** (4.5)	0.41		
***S. haematobium* urine eggs** (median,	**0** (0-0)	**12** (4-63)	**<0.001**		
**Other helminths (%)**	**0**	**17.9**			
***Ascaris lumbricoides* (%)**	**0**	**2.6**			
***Trichuris trichiura* (%)**	**0**	**10.3**			
***Necator americanus* (%)**	**0**	**7.7**			
Strongyloides (%)	**0**	**2.6**			
**TIgE (IU/L)** (median, IQR)*	**6216** (1545-	**10476** (6570-	**0.034**	**3226** (-1584, 8035)	0.19
**Eosinophils (%)** (mean, SD)*	**10.3** (8.2)	**18.0** (9.6)	**0.011**	**7.1** (0.9, 13.4)	**0.025**
**hs-CRP (mg/L)** (median, IQR)	**1.91** (0.49-4.52)	**1.71** (0.68-4.07)	0.78	**-0.12** (-0.81, 0.56)	0.72
**ALAT (GPT, U/L)** (mean, SD)	**19.2** (11.9)	**17.1** (9.3)	0.42	**-3.3** (-8.4, 1.8)	0.20
**ASAT (GOT, U/L)** (mean, SD)	**26.4** (10.1)	**24.2** (7.0)	0.29	**-2.9** (-7.0, 1.2)	0.16
**Glucose (mmol/L)** (mean, SD)	**4.61** (1.06)	**4.52** (0.67)	0.66	**-0.14** (-0.57, 0.28)	0.51
**Insulin (mU/L)** (median, IQR)	**4.45** (2.74-7.21)	**4.54** (2.91-9.62)	0.91	**0.06** (-0.43, 0.55)	0.81
**C-peptide (nmol/L)** (median, IQR)	**0.41** (0.32-0.61)	**0.36** (0.27-0.62)	0.59	**-0.05** (-0.38, 0.27)	0.75
**HOMA-IR** (median, IQR)	**0.94** (0.52-1.32)	**1.00** (0.57-1.80)	0.87	**0.06** (-0.45, 0.58)	0.80
**QUICKI** (mean, SD)	**0.40** (0.08)	**0.40** (0.08)	0.96	**-0.00** (-0.04, 0.04)	0.92
**TC (mmol/L)** (mean, SD)	**4.42** (0.84)	**4.01** (0.81)	**0.037**	**-0.30** (-0.68, -0.08)	0.11
**HDL-C (mmol/L)** (mean, SD)	**1.44** (0.40)	**1.18** (0.31)	**0.003**	**-0.24** (-0.43, -0.06)	**0.009**
**LDL-C (mmol/L)** (mean, SD)	**2.56** (0.73)	**2.50** (0.78)	0.74	**-0.04** (-0.31, 0.38)	0.84
**TG (mmol/L)** (mean, SD)	**0.93** (0.50)	**0.72** (0.21)	**0.031**	**-0.20** (-0.39, -0.03)	**0.022**

Results

Reviewer #1:

Results are clearly and completely presented. One aspect not addressed is the relationship of the immune response (as determined by eosinophils numbers and or %) to the lipid profile for each individual. The authors do mention in the discussion that the possible effect of IL4/IL13 on hepatocyte function may be one of the mechanisms for lowering TG. Eosinophil levels do reflect the immune response to the parasite by an individual and although the authors are correct to use CAA levels to measure intensity of parasite infection it would also be worth it I thought to measure intensity of the immune response to the Sh and lipid levels.

Authors' reply:
We do agree with the reviewer that his/her suggested analysis would have make sense and nicely complement the one done using CAA. Unfortunately, as acknowledged, part of the eosinophil data are missing due to lost samples during field analysis and, as such, we do have a reduced statistical power (especially when the data are stratified according to BMI), preventing to draw reliable and firm conclusions. Of note, when doing this analysis for the whole population (see Figure 1 for reviewers below), some similar trends than the ones observed with CAA are still observed but none of them reached statistical significance due to low sample size.

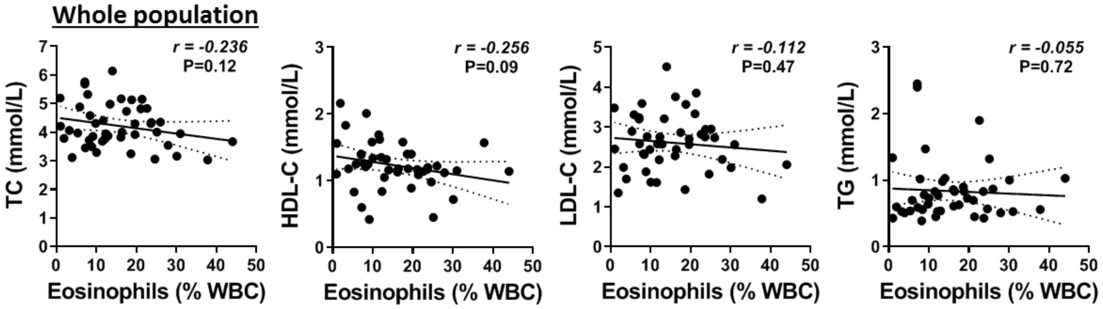

Figure 1 for reviewers: Associations between intensity of S. haematobium infection assessed by blood eosinophil levels and serum lipid parameters in the whole population

The 'Statistical Analysis' paragraph is well written. These are a good choice of tests, consideration of confounding factors, and multiple testing corrections. Tables S1 & S2 is where they show the raw Odds Ratio and confounder adjusted OR for egg and serum levels. Tables S1, S2 are very informative, and are well presented by showing both raw and adjusted results (that consider multiple important confounding factors). Tables S3, S4, S5 are mislabelled.

Authors' reply:
The labels of Tables S3-5 have been corrected (see new Tables).

Table S3 summarises factors stratified by CAA range; the Eosinophil response appears to show incredibly strong correlation to stratification level, it is unfortunate they lost some measurements for Eosinophils as stated, but their choice of statistical test is well suited to different sample sizes. Table S4 shows the disparity between gender between BMI >/< 25 groups, showing the importance of showing adjusted OR – It is important that the presentation of raw data is included. Fig. 1 It is interesting when whole population the HDL-C has a significant p-value but when you split it into lean & obese there is no significant p-value in either, despite the same trend. The overall trend is clear however that serum CAA levels are associated with many cholesterol measurements in the obese category. Fig. 2 a and b are skillfully plotted - and a statement is needed with respect to what p-values are associated to (#/*/#*) on the heatmap.

Authors' reply:
The definition of the p-value labels has been added to the legend of Figure 2 (Page 23)

Reviewer #2:
The results are well presented and clear

Reviewer #3:
The CAA corrected data should be included in the main paper and not as supplemental materials. The authors should simplify the results and use only the CAA positive and CAA negative as the Sh+ and Sh- groups in the paper. Table 2 should be corrected using the CAA data.

Authors' reply:
We respectfully underline that in the framework of our field study Sh infection was initially determined by urine egg detection, and as such, these data should be reported. Later on, we refined our analyses by determining serum CAA levels and used these results to stratify the data based on infection intensity. All the CAA-related analyses are currently present in the manuscript, mostly in the main Table/Figure (Table 3, Figures 1 & 2). We think that the way we are presenting our data is currently appropriate, in line with the other reviewers' comments.

Figure 1: Please include the number of patients for each analysis (whole population, BMI<25, and BMI>25).

Authors' reply:
This information has been added to the legend of Figure 1 (Page 23)

Figure 2: Please include the definition of * and # in the figure legend.

Authors' reply:
The definition of the p-value labels has been added to the legend of Figure 2 (Page 23)

Conclusions

Reviewer #1:
The conclusions are supported by the data and the authors have been clear about the limitations of the data. The authors discuss well the relevance of the data to human health as it relates to lipid levels in individuals with high BMI. Of course it is a big jump from this study to proposing Sh infection would have similar effects on Dutch people who have never previously encountered Sh infection.

Authors' reply:
We do agree with the reviewer and that's why we first suggest using larger cohort and/or performing deworming intervention (line 301-303, Page 15).

Reviewer #2:
The authors have interpreted their results and limitations well, highlighting where their findings fit with the (rapidly growing) literature in this field relating to worms and metabolism. There remains obvious mechanistic unknowns as to HOW Sh regulates metabolism, some more in depth analysis of cytokines, cellular immune responses and the microbiome would have been useful to understand this, but this could form the basis of future work (and is well discussed anyway).

Authors' reply:

We fully agree with the reviewer and, although we speculated in the discussion on some possible underlying mechanism(s), further studies are definitely required for improving our understanding.

Reviewer #3:
I recommend the authors to include a short conclusion paragraph at the end of the discussion. Paragraph 273-292: The article by Cortes-Selva D et al. (Frontiers in Immunology 12;9:2580) should be included since it reinforces the hypothesis that Schistosoma induced Th2 response confers protection from hyperlipidemia, atherosclerosis, and glucose intolerance.

Authors' reply:
Together with a short sentence, we have added the suggested publication in the discussion section (line 297-298, Page 15). It might indeed support changes in tissue-resident immune cell lipid/cholesterol metabolism in response to helminth infection.

Editorial and Data Presentation Modifications?

Reviewer #1:
The 'Statistical Analysis' paragraph is well written. These are a good choice of tests, consideration of confounding factors, and multiple testing corrections. Tables S1 & S2 is where they show the raw Odds Ratio and confounder adjusted OR for egg and serum levels. Tables S1, S2 are very informative, and are well presented by showing both raw and adjusted results (that consider multiple important confounding factors). Tables S3, S4, S5 are mislabelled. Table S3 summarises factors stratified by CAA range; the Eosinophil response appears to show incredibly strong correlation to stratification level, it is unfortunate they lost some measurements for Eosinophils as stated, but their choice of statistical test is well suited to different sample sizes. Table S4 shows the disparity between gender between BMI >/< 25 groups, showing the importance of showing adjusted OR – It is important that the presentation of raw data is included. Fig. 1 It is interesting when whole population the HDL-C has a significant p-value but when you split it into lean & obese there is no significant p-value in either, despite the same trend. The overall trend is clear however that serum CAA levels are associated with many cholesterol measurements in the obese category. Fig. 2 a and b are skillfully plotted - and a statement is needed with respect to what p-values are associated to (#/*/#*) on the heatmap.

Authors' reply:
See previous response in the Results section

Reviewer #2:
(No Response)

Reviewer #3:
As mentioned above, the tables presented in the main text should be modified to include the CAA data. CAA text is more sensitive and should be considered as the "true positive and true negatives." The analysis based only on the direct observation of eggs in urine does not need to be included in the main text since it contains false negatives.

Authors' reply:

See previous response in the Results section

QUICKI should be calculated and included in the paper.

Authors' reply:
See previous response in the Results section

Summary and General Comments

Reviewer #1:
This is an interesting and provocative study. I do think it would be interesting to put in a comparison of all the individual immune responses to Sh (as measured by eosinophil levels) and the effect on lipid levels.

Authors' reply:
See previous response in the Results section

Reviewer #2:
I think this is a timely, novel and interesting study. Some clarification as to the relative importance of S hematobium versus the other co-endemic helminths would add important supportive evidence

Authors' reply:
See previous response in the Results section

Reviewer #3:
Zinsou et al. observed that overweight/obese individuals infected with Schistosoma haematobium have an improved lipid profile. This manuscript is relevant to the readers of PlosNTDs, and this study is the first report that evaluated the impact of a helminth infection in overweight/obese individuals from endemic areas. The main limitation of the study is the sample size. The authors need to inform how the sample size was calculated.

Authors' reply:
See previous response in the Methods section

The manuscript is well written, but the data could be better presented. The Sh+ and Sh- groups should be divided based on CAA test since it is more sensitive than the parasitological exam (presence of eggs in urine). However, the authors present this data mostly as supplemental material.

Authors' reply:
See previous response in the Results section
